# Catalytic multi-step domino and one-pot reactions

**DOI:** 10.3762/bjoc.20.25

**Published:** 2024-02-08

**Authors:** Svetlana B Tsogoeva

**Affiliations:** 1 Department of Chemistry and Pharmacy, Organic Chemistry Chair I and Interdisciplinary Center for Molecular Materials (ICMM), Friedrich-Alexander-Universität Erlangen-Nürnberg, Nikolaus Fiebiger-Straße 10, 91058 Erlangen, Germany

**Keywords:** domino reactions, multi-step reactions, multicomponent reactions, one-pot synthesis, organocatalysis, tandem reactions, transition-metal-catalysis

The synthesis of pharmaceutical ingredients, natural products, agrochemicals, ligand systems, and building blocks for materials science has reached a high level of sophistication over the past decades. Most known processes, however, are still frequently hampered by lengthy protecting-group strategies and very costly purification procedures derived from the "stop-and-go" synthetic methods (Figure 1a). Those protocols are still far from the ideal synthesis, implying high atom efficiency, step and pot economies, decreased number of purification steps, or protecting-group-free synthesis.

**Figure 1:**
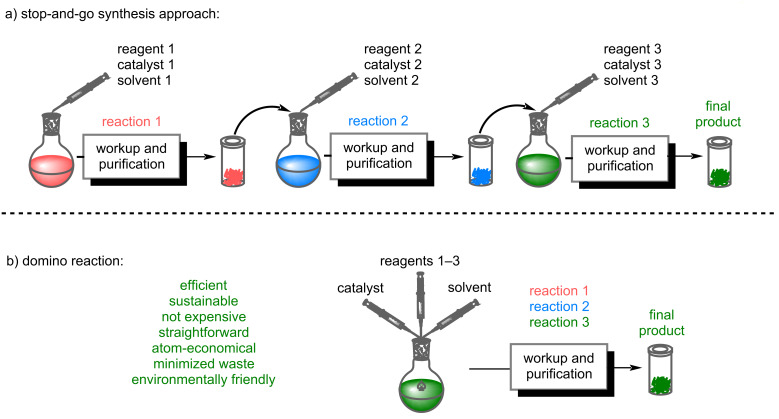
Comparison of a classical “stop-and-go” synthesis with a domino reaction.

Multi-step domino [1–2] and one-pot [3] reactions represent a new powerful toolbox in organic synthesis to install molecular complexity economically and sustainably, starting from simple compounds. In particular, catalytic domino reactions and one-pot processes with excellent selectivity and functional-group tolerance are of significant interest to industrial and academic research. In the so-called domino reactions, all of the required reagents, the catalyst, and a solvent are mixed in one flask, in which all reaction steps take place autonomously without the addition of further reactants [1]. The domino process contrasts the conventional "stop-and-go" synthetic approach in the way that multiple reactions proceed in a direct sequence. In such a reaction sequence, one step triggers the next, which in the end yields a complex product. In contrast to a conventional “stop-and-go” method, only a single workup and purification is needed (Figure 1b), and therefore these reaction cascades can be considered superior to stepwise synthetic approaches in the context of green chemistry because they are time-saving, waste-reducing, and atom efficient [1–6]. These efficient and straightforward synthetic methods make the isolation and purification of intermediate products after each reaction step superfluous, thereby drastically reducing the number of workup and purification steps, which is superior to conventional organic synthesis.

The Thematic Issue “Catalytic multi-step domino and one-pot reactions” in the *Beilstein Journal of Organic Chemistry*, which I had the pleasure to edit, covers the recent strategies of domino reactions and one-pot syntheses and presents the advances in the field. A Review article by Pounder, Tam, and co-authors summarizes new transition-metal-catalyzed domino reactions of strained bicyclic alkenes, including both homo- and heterobicyclic alkenes highly useful for the construction of biologically significant compounds with multiple stereocenters [7]. In the Review paper by Kisszékelyi and Šebesta, the diverse variety of chiral metal enolates obtained by asymmetric conjugate additions of organometallic reagents and the possibilities to engage metal enolates in tandem reactions with new electrophiles are presented [8]. A Perspective from X. Zhang, Ma, and W. Zhang reflects the state of the art in the α-amino acid-based [3 + 2] cycloaddition reactions of N–H-type azomethine ylides in multicomponent, one-pot, and stepwise reactions for the synthesis of diverse bioactive heterocyclic compounds and natural products [9].

Computational studies of transannular cycloadditions of cycloalkenone hydrazones catalyzed by BINOL-derived phosphoric acid are reported in the Full Research Paper by Vicario, Merino, and co-workers [10]. Their methodologies can now be used to predict the reactivity of different substrates in other cycloaddition reactions through concerted or stepwise mechanisms. An enantioselective palladium-catalyzed three-component reaction of glyoxylic acid, sulfonamides, and aryltrifluoroborates toward synthetically useful α-arylglycine compounds is described by the Manolikakes group [11]. Moreover, Šebesta and co-workers report a facile stereoselective tandem reaction based on the asymmetric conjugate addition of dialkylzinc reagents to unsaturated acylimidazoles, followed by trapping of the intermediate zinc enolate with carbocations [12]. A practical one-pot synthesis of fluorescent pyrazolo[3,4-*b*]pyridin-6-ones by reacting 5-aminopyrazoles with azlactones under solvent-free conditions, through subsequent elimination of a benzamide molecule in a superbasic medium, is described by the Fisyuk group [13]. A further facile one-pot process toward a new series of copper(II) benzo[*f*]chromeno[2,3-*h*]quinoxalinoporphyrin analogues is described in the Full Research Paper by Nath and co-workers. The newly synthesized porphyrin derivatives displayed significant red-shifted absorption and emission compared to simple *meso*-tetraarylporphyrins [14]. The Carlone group reports an Enders-type triple cascade reaction toward cyclohexenals, using acetaldehyde dimethyl acetal as a masked form of acetaldehyde, which is hydrolyzed in situ, allowing for a higher product yield and fewer byproducts [15]. The group of Müller describes an elegant consecutive four-component reaction involving an alkynylation–cyclization–iodination–alkylation sequence toward trisubstituted 3-iodoindoles, which are valuable substrates for the synthesis of, e.g., blue emitters in good yield [16]. The power of double click reactions toward functionalized bis(1,2,3-triazole) derivatives has been demonstrated in the Full Research Paper by Reissig and Yu. The authors successfully combined nucleophilic substitution of benzylic bromides with sodium azide and a subsequent copper(I)-catalyzed double click reaction in one pot [17].

In summary, these contributions by renowned experts demonstrate the broad diversity of impressive catalytic domino, tandem, and one-pot processes towards many valuable compounds.

Finally, I would like to take this opportunity to warmly thank all the authors of this thematic issue for their beautiful contributions. I also sincerely thank all the referees and the Editorial and Production Teams of the *Beilstein Journal of Organic Chemistry* for their highly professional assistance and support.

Svetlana B. Tsogoeva

Erlangen, January 2024

## Data Availability

Data sharing is not applicable as no new data was generated or analyzed in this study.
